# The Foreign Journals: Anatomy and Physiology

**Published:** 1841-07

**Authors:** 


### PART THIRD.

### Selections from the British and Foreign Journals.

### I. THE FOREIGN JOURNALS.

### ANATOMY AND PHYSIOLOGY.

*On the respective Functions of the Upper and Lower Halves of the Spinal Cord, and their relation to the Extensor and Flexor Muscles of the Limbs.* By Ed. Engelhardt.

If, after cutting off the head of a frog, a wire be pushed slowly from above downwards through the vertebral canal, the two thighs are drawn forcibly to the abdomen, and the feet are made to meet above, and are pressed against the wire; in short, motions of the legs take place which have exactly the appearance of being intended to put away the wire. If the wire be now carried farther into the spinal marrow, the same motions still, for a time, continue to be produced, though less forcibly than before; but, when it has been pushed to about the middle of the vertebral column, they assume all at once a different character, and the legs are no longer drawn up to the abdomen, but are forcibly thrust away from it. The deeper the wire is passed, the more powerful do these movements of extension become; nor do they cease till the very last portion of the spinal marrow is destroyed.

To determine more exactly the spot at which irritation of the spinal marrow ceased to produce flexion, and brought on extension of the legs, I instituted the following experiments on a number of frogs: I cut off the head with scissors; and at the instant the legs were every time drawn up forcibly towards the abdomen, and the feet pushed against the scissors. I then removed the abdominal and thoracic organs, laid the trunk on its back on a smooth board, placed the legs in a half-flexed position, and cut through portions of the spinal marrow at each vertebra successively, proceeding regularly from above downwards. The legs were every time, as soon as the motions produced in them by each section had ceased, again placed in the half-bent posture before another section was made. The result was as follows: from the first to the fourth vertebra, the sections of the cord produced flexions of the thighs and stretching out of the feet over the upper part of the trunk. The more there was cut off from the cord, the weaker were these movements; and, when the sections came to be made at the part between the fourth and fifth vertebra, the legs were no longer drawn up, but movements of extension were produced in them, which increased in force in proportion as the sections were made nearer to the os coccygis. When the whole spinal marrow was thus removed, and portions were at the same time cut off from the three nerves which compose the sacral plexus, the result was, that only movements of extension of the legs were produced; and these continued till the section (carried onwards by degrees) came to be made at the part where the plexus divides into the ischiatic and the crural nerves.

To see whether the anterior limbs were similarly influenced by stimulating particular portions of the spinal marrow, I modified the experiment in the following manner: after cutting off the head, (by which the fore-legs were thrown into violent contractions,) I removed the abdominal and thoracic organs, without injuring the walls of the chest or the muscles of the shoulder attached to them. I then divided the vertebral column between the eighth vertebra and the sacral vertebra, and pushed a wire from below into the vertebral canal. The result was, that, while the lower half of the spinal marrow was being destroyed, the anterior limbs were extended; but as soon as the wire reached the upper half of the cord they were flexed.

To test the influence of division of the upper and lower halves of the cord respectively on the reflex motions, I repeated the above experiments on several other frogs; and after replacing the legs in the half-bent position, as soon as the motions produced in them by the division of each vertebra and the cord within it had ceased, I stimulated the toes of one of the feet by pinching them with forceps. I now observed, that so long as the cord was not cut away by the successive sections, so far as the space between the fourth and fifth vertebræ, movements of flexion only were produced in the irritated leg, and in that opposite to it. But if the division were made at *this* spot, then irritation of the toes of one foot no longer produced flexion either in the corresponding or the opposite leg, but in several cases excited extension, which, however, was weaker than when the marrow itself was directly stimulated. As soon as the spinal marrow was divided between the fifth and sixth vertebræ, the reflex motions ceased, though direct stimulus of the marrow even down to the sacral vertebra still produced the same extension of the hind-legs as in the first-mentioned experiments.

Now, as the vertebral column of the frog consists of eight free vertebræ and one sacral, and division of the spinal marrow from the medulla oblongata down to the fourth vertebra produces flexion of the limbs, and from the fifth vertebra to the end of the column produces extension; and since, moreover, the experiments on the alterations of the reflex motions, according as the marrow is divided in its upper or in its lower part, produce a result which exactly agrees with these, it appears to follow that there is a determinate antagonism between the functions of the upper and the lower half respectively.

*Muller's Archiv. Heft* 3. 1841.

---

### *On the Anastomoses of Nerves.* By A. W. Volkmann.

Under this name the author includes those cases in which the branch of one nerve is connected with the trunk of another, and passes in the latter, not downwards but upwards, and towards the nervous centre; an arrangement similar to that of the terminal loops, but occurring in these cases, not in isolated fibres, but in whole branches.

One loop of this kind exists frequently, if not regularly, between the fourth pair of nerves and the first division of the fifth in the calf. Looking at it with the naked eye, in one case the loop appeared to consist of a single filament; but, on examining it with the microscope, five branches were found to arise from it, of which four received their fibres from the fourth, and only one from the fifth. This last was fully ten times smaller than that part of the loop which was connected with the central portion of the fifth. If, therefore, this portion were considered as coming from the fifth, there would be only one tenth of the fibres going to the periphery, while nine tenths must have been passing to the brain within the sheath of the fourth nerve. For, since the fourth above the loop gave off no branches, there was no opportunity for these fibres to turn round out of it again, and pass to the periphery. The character of the loop here assumed can therefore be doubted only by supposing that the portion, which seemed to come from the fifth, was really a branch passing into it; in which case it must have consisted of fibres of the fourth passing centripetally in the fifth. Such fibres might thus pass to the Gesserian ganglion, and thence go peripherally in some other branch of the fifth pair.

A second loop of the same kind appears to exist pretty generally in mammalia, between the second or third cervical nerve and the accessory. The author has found it in men, horses, dogs, calves, and cats. In the calf, the anterior branch of the second cervical gives off a considerable branch, which goes to the deep main branch of the accessorius. One portion of its fibres passes within the accessorius, towards the periphery; the other portion, centripetally. Above their union, branches are given off from the accessorius, so that there is an opportunity for the centripetal fibres just mentioned to pass out of it again, and proceed centrifugally; but the microscope shows that they do not do this. Or, again, as the vagus and the accessorius are connected at the ganglion on the former, the fibres in question might go into the vagus, and then proceed peripherally in it; but, in the first place, the author once found the loop where it was evident that the vagus and accessorius only lay close by each other, without mixing fibres; and, in the second place, he was fortunate enough on one occasion to trace a fasciculus of fibres from the loop of the cervical completely into the root of the accessorius without a break. With the microscope and compressorium, he convinced himself that the fibres thus actually passed through the accessorius into the brain. Unless, therefore, it were admitted that the anastomosis between the accessorius and the second cervical connects two portions of the central organ, there remains only the hypothesis, that the fibres of this loop belonged to the accessorius, and that they ran at first centrally in the cervical nerves, but at one of its branches turned round, and proceeded centrifugally. But experiments proved (as will be shown) that at least a portion of the fibres were sensitive, and came from the spinal cord.

A third and similar loop is found between the cervical nerves and the descending branches of the hypoglossal. In man, as well as in dogs, rabbits, sheep, lynxes, calves, and cats, connecting branches of the cervical nerves pass into the descendens noni, and in it some of their fibres proceed peripherally, but a larger portion of them go towards the nervous centre. These last fibres pass on to the tongue,* and it is remarkable that what is called the descendens noni appears to contain only a few fibres from the ninth nerve. Excluding those fibres in the descendens noni, which, as they go to the tongue, cannot be supposed to come from the ninth, there is the highest probability that many of the fibres which certainly do come from the ninth instead of remaining in the descending branch, go through a branch of the uppermost cervical nerves to the spinal cord.

The author believes that he has obtained a proof of this by dissection of the nerves in the sheep. In it the descendens noni, where it comes off, forms a plexus of numerous fibres, which if it be carefully cleaned, and placed on a glass plate beneath the microscope, permits all its fibres to be closely traced. It is plain that, in this case, far more fibres pass to the descendens noni from the trunk of the ninth than it contains below the part where it is connected with the cervical nerves; and it thence follows, that a part of the fibres which pass from the ninth into its descending branch, do not remain in it, but go through the cervical to the cord; and this is the more probable, because these fibres could not again pass out of the cervical nerves to go peripherally, except at a very acute angle; and there are no signs of such an arrangement.

A fourth anastomosis exists between the second and third cervical nerves of the cat. The third, immediately at the origin of the anterior branch, gives off a large branch which first forms the loop already mentioned with the accessorius, but then passes completely into the second cervical nerve, in which a considerable portion of its fibres run backwards to the nervous centre. Between the first and second cervical nerves of the cat, there is also a constant communication, which, however, when closely examined, has rather the appearance of a plexus.

From what has been said, it results that there are nervous loops in which the fibres are placed together, but from which they do not go to be distributed in

* See paragraph 43, in the paper "On the Motor Influences of the Cerebral and Cervical Nerves."

one organ. Such an arrangement must certainly be considered strange; but its strangeness is of the less weight against its reality, from its being exactly analogous to the terminal loops. The union of adjacent fibres (such as occurs in the terminal loops) is in no respect more intelligible than that of the fibres of distinct nerves; and indeed experiments on these larger loops may serve to illustrate the infinitely more delicate terminal loops, to which experimental investigation is inapplicable. Such experiments were therefore performed, and their results follow:

The skin was divided behind and below the transverse process of the atlas of a live calf, and the anterior branch of the second cervical nerve was exposed. It had already divided into two branches, of which one was directed backwards and downwards, the other forwards and downwards. The latter contained in a looser cellular sheath the finer branch, which lower down became free, and was connected with the accessorius. Around this anterior branch, an inch and a quarter below its exit from the vertebral column, a ligature was tied, producing to all appearance at the instant acute pain. A second ligature was then applied below the former, and therefore nearer to the accessorius. This second ligature was at first tied only gently, but the animal gave out manifest signs of pain. After a short pause, the knot was pulled quite tight, and again there was distinct evidence of pain, though the animal seemed to suffer less than when the first ligature, which was nearer to the origin of the cervical nerves, was applied.

The anastomosis between the cervical and the accessorius was exposed in a calf, and was found to consist of two separate filaments. On tying the first pain was produced, but a second ligature tied on it nearer to the accessorius excited no appearance of pain. Three ligatures were then placed one close behind another on the second branch. The first, which was put in the middle of the branch, gave severe pain; the effect of the third, which was applied close to the union with the accessorius, was doubtful, the animal only drew back as if hurt. Supposing pain was produced, it must have been excited through branches of the accessorius, some of which other subsequent experiments proved to contain sensitive fibres.

The loop between the cervical and accessory nerves was exposed in a calf, and with short pauses after each application was tied three times. The first ligature excited pain, so did the second, which was placed nearer the spinal cord; but so did not the third, which was applied nearer to the accessorius than the first was. A fourth ligature was applied on the accessorius itself, and gave distinct pain.

The accessorius was exposed in a calf near its exit from the skull, and tied above its connexion with the second cervical nerve. The animal struggled and bleated during the drawing of the knot; after a pause the nerve was divided above the ligature, but there was no sign of pain. Then the divided nerve was repeatedly irritated at its peripheral end, and distinct indications of suffering were noticed every time.

Neglecting the very slight anomalies that were observed, and which may fairly be referred to the difficulty and tediousness of the operations, these experiments appear clearly to establish, 1st, that the anastomosis connecting the accessorius with the second cervical nerve is sensitive; 2d, that the course of conduction of the nervous influence is twofold, stimuli producing sensation by acting towards the brain as well as towards the spinal cord; 3, the sensitive fibres in the loop that belong to the spinal cord are considerably predominant.

The anastomosis between the second and third cervical nerves in a cat was exposed. A ligature tied in the middle of the loop excited the severest pain. A second ligature applied close to the part where the loop seemed to come out from the second cervical nerve produced no reaction. A third placed close to the third nerve gave acute pain. The same experiment was repeated with the same result in another cat. In a third the loop was divided close to the second nerve, and on irritating the portion of it nearest to the third nerve, there were some signs of pain, but the animal was too much exhausted to draw any strict deductions from its appearance of suffering.

[Other experiments were tried with the other anastomoses, but the author confesses that the difficulties of tracing such minute fibres as compose these nerves in the living subject, were too great to permit his attaining any definite result. He then enters upon a theoretical consideration of the intentions served by these anastomoses; but into this, as his hypothesis seems very far less valuable than his facts, it does not appear necessary to follow him.]

*Müller's Archiv*, 1840. *Heft* v. *p.* 510.

---

### *On the Functions of the Roots of the Nerves.* By M. LONGET.

LAST year M. Longet addressed the Royal Academy of Medicine, deducing from certain experiments the inference that the anterior or motor root of the spinal nerves was endowed in a certain degree with the faculty of sensation, and that it owed this faculty, not to its relations with the antero-lateral column of the spinal marrow, but those which it has, by means of the spinal ganglion, with the corresponding posterior root. M. Longet has since repeated and modified these experiments, and finds, after trials upon seventeen dogs, and upon the roots of ten nerves in each dog, (thus making 170 experiments), that, uniformly, the anterior roots and the corresponding columns of the spinal marrow are insensible to every kind of mechanical irritation, while the posterior roots and the posterior spinal columns are always exceedingly sensitive. "But," he adds, "in applying alternately the two poles of a voltaic pile of twenty couples to the two roots under similar circumstances, I excited the most violent convulsions in acting on the anterior roots, while not the slightest trace of convulsions was manifested in experimenting on the posterior roots." In all these experiments the roots were isolated by plates of glass. Whether mechanical irritation, then, or galvanism be employed, the phenomena are so uniform and so evident, that we cannot doubt that the anterior roots are exclusively motor, and the posterior exclusively sensitive.

Galvanism may pass from the anterior column of one side to that of the opposite side, through the medium of the anterior white commissure which unites them; but it is worthy of remark, that it never passes from the posterior to the antero-lateral column through the medium of the posterior corner of gray substance which completely separates these two columns. The gray substance, then, appears to be a bad conductor of electricity.

*Journal des Connaissances Médico-Chirurgicales. Février*, 1841.

---

### *On the Motor Influences of the Cerebral and Cervical Nerves.* By A. W. VOLKMANN.

IN experiments on the motor influences of the cerebral nerves, it is necessary to irritate their roots within the skull, because connexions take place between their branches soon after their exit from it, and sometimes even within its cavity. For this purpose the author adopted the plan of making longitudinal sections of the heads of fresh-slain animals, and irritating the nerves within the skull, either mechanically, or with the wires of a galvanic battery of which he could regulate the force. [We need not detail the cautions adopted to shut out all sources of error; the reputation which Volkmann has already earned in this branch of physiology is the best guarantee of the correctness of his experiments, and of the care with which he would draw results from them.]

I. *Of the oculo-motor or third pair of nerves.* 1. In a fresh-slain calf, the orbit was broken into and the muscles of the eye exposed. On irritating the root of the third nerve within the skull, contractions ensued in the levator palpebræ, rectus superior, rectus internus, rectus externus, obliquus inferior, obliquus superior, and retractor bulbi.* Repeated experiments on several calves, a cat and a sheep, always gave the same results. In the sheep the experiment was performed so as to remove all suspicion that the motions of the obliquus superior and rectus externus were secondary. The orbit was broken into, and the eyeball with all its muscles, except these two, removed; but the irritation of the oculo-motor nerve still excited contractions in them both, though in the obliquus superior much more violently than in the rectus externus.

2. A dog's skull was sawn through longitudinally, the orbit broken into, and all the recti muscles cut away directly after death. On irritating the third nerve, the direction of the pupil was not in the least altered; the eye did not move upwards, but it turned round on its axis just like a wheel on its axle-tree. The upper portion of the sclerotica described a small portion of a circular arc, and approached the outer angle of the eyelids. This experiment was repeated with a slight modification in another eye. The lower eyelid, and a portion of the lower margin of the orbit, were removed from a fresh-slain dog, the exposed obliquus inferior was irritated, and the result was the same; the rotation was like that of a wheel, and as regular as if the visual axis were so fixed as to prevent any other movement of the eye except this of rotation. The experiment was repeated and confirmed in the calf.

3. The healthy eye of a live calf was observed, and Bell's idea that at every wincing the pupil suddenly moves upwards and inwards, was found as little confirmed here as it is by examining the eyes of the rabbit, horse, and cat. A rotatory motion, however, was often noticed; the elliptical iris, with its long diameter, going almost transversely across the eye, moved round the pupil like the beam of a balance round its pivot, so that the posterior (external) end of the ellipse was considerably depressed, and the anterior as much elevated. The obliquus inferior was now exposed and divided, and the balance-like motion of the iris was no longer perceptible. When the conjunctiva was irritated, there was often no motion of the eyeball; sometimes, however, there was a sudden retraction of it, and sometimes a twitching of it upwards.

II. *Fourth pair.* 4. In a fresh-slain calf prepared as before, the fourth nerve was irritated within the skull. The eye turned round on its axis, and the balance-movement of the iris was manifest, but its direction was the reverse of that in exp. 3, the anterior part being depressed and the posterior raised. The pupil was not directed outwards and downwards. In the cat the same phenomena occurred, but in a less degree.

5. The orbit of a fresh-slain animal was broken into, and the fourth nerve galvanized. The exposed obliquus superior contracted, but it did so in like manner when the third nerve was irritated (exp. 1.) Endeavours to make other muscles move through the medium of the fourth nerve were uniformly unsuccessful.

III. *Sixth pair.* 6. On irritating this nerve after similar preparation of the head and orbit, violent contractions ensued in the exposed rectus externus, drawing the eye outwards, and producing various other twitchings. After removing this muscle, and irritating the sixth nerve, there ensued twitching movements of the eyeball, and the pupil was drawn into various directions. After removing all the other muscles, the retractor bulbi was found acting as often as the nerve was irritated; and according as one or other of its fasciculi contracted, the pupil was drawn into this or that direction. In no experiment could the inferior oblique be excited through the sixth nerve.

7. In another similar case irritating the sixth nerve excited irregular motions in different directions, and especially a turning of the pupil outwards; and every time the nervewas irritated, the third eyelid was moved from the inner towards the outer angle of the eye.

* "My assistant has traced the branches of the third nerve to these last two muscles. The third in the calf gives a branch to the fourth nerve."

IV. *Fifth pair.* 10*. In a fresh-slain calf, the smaller root of this nerve was galvanized, and violent motions of the jaws ensued. The same result was obtained in other animals. When the great root of this nerve, or the root of any other of the cerebral nerves was irritated, no motions of the jaw took place. The muscles of the lower jaw of a calf were exposed, and irritation of the small portion of the fifth nerve was then seen to produce contractions in the mylo-hyoideus, anterior portion of the digastricus, temporal, masseter, and internal pterygoid. The external pterygoid could never be exposed while the irritability lasted. On irritating the small portion of the fifth, neither the buccinator nor the soft palate was ever moved; the author believes, therefore, that though Bell, on anatomical grounds, attributed to this nerve the motions of the buccinator muscle, and Valentin on similar evidence those of the soft palate, its motor influence is really limited to the muscles enumerated above.

V. *Seventh pair, or facial.* 11. In calves, dogs, sheep, and goats, the root of the facial being exposed and galvanized, distinct contractions occurred in the following muscles: frontalis, buccinator, orbicularis palpebræ, orbicularis oris, part of a muscle moving the nose, and of another moving the angle of the mouth, the attrahens, retrahens, and attollens auris, the posterior belly of the digastricus, the stylo-hyoideus and platysma.

12. The author could never succeed in exposing the muscles of the tympanum so as to see them act, but in his attempts he several times found irritation of the chorda tympani produce contractions of the buccinator.

13. On irritating the portio intermedia Wrisbergi, twitchings ensued in all the muscles of the face and ear, in the same manner as if the great portion of the facial had been irritated.

14. In a great number of experiments, irritation of the facial never produced motions of the tongue. 15. Neither is the soft palate ever moved by the influence of the facial, either in sneezing, as Bidder supposed, or in swallowing, as Valentin held. In the cases in which such motions seem to occur, the movements of the palate are only secondary to those of the tongue. The facial governs the digastricus and stylo-hyoideus; and when these are divided, or their action has ceased, the motions of the soft palate are no longer discernible, nor can they ever be excited, though all the muscles of the face and ears are thrown into active contractions. It is therefore manifest that the branch of the facial, which, under the name of superior vidian, passes to the sphino-palatino ganglion, does not convey any motor filaments to the soft palate.

VI. *Glosso-pharyngeal.* 16. This nerve arises in the calf, dog, cat, and man, by two distinct roots, each of which may be separated into two fasciculi. On the larger root (and often even within the skull) lies the Ehrenritter ganglion. But this does not seem to be really distinct from the petrous ganglion; for often only one ganglion can be found, and when there appear to be two, they are always connected by a tract of ganglionic substance. The separation of the two is therefore accidental; and however varied their apparent arrangement, it may be stated as a general rule, that all the fibres of the glosso-pharyngeal nerve pass through ganglionic matter. It is impossible therefore to compare the two roots of this nerve with those of the spinal nerves, or the Ehrenritter ganglion with the ganglia of the spinal sensitive roots.

17. After numerous unsuccessful attempts to produce motion by irritating this nerve, the author met with a case in which its two roots were separated, not only from the vagus, but from each other by dura mater. Irritation of the larger of these roots produced no effect, but that of the smaller (which in the previous experiments had been overlooked) excited violent motions in the pharynx. On exposing the latter it appeared that the middle constrictor and the stylo-pharyngeus were acting. This result was confirmed on two calves and

* For convenience of reference, the numbers of the paragraphs are marked as in the original paper; a considerable portion on the actions of the muscles of the nerve is here omitted, but is printed in a separate abstract.

two cats, so as fairly to establish, 1st, that the glosso-pharyngeal nerves govern only these muscles; 2d, that no other nerve has any influence on them; and 3d, that only the small root of the nerve is endowed with motor power. In the experiments of Valentin and John Reid, who could find no direct motion produced by irritating the glosso-pharyngeal, its smaller root must have been overlooked, as it was by the author in his first experiments.

VII. *Nervus vagus.* 18. In the dog and sheep a part of the roots of the vagus pass by the ganglion, and are connected with it only by cellular tissue; but in the calf this is not the case; several fibres seem indeed to go behind the ganglion, but on these there are minute irregularities, which when examined with the microscope are found to be true ganglionic matter. All the fibres in the calf therefore pass through ganglionic matter; a fact which is of some importance in relation to the following experiments, and to the physiology of ganglia and gangliated nerves.

19. A fresh calf's head was separated from the trunk and sawn longitudinally, so that the soft palate and upper part of the pharynx could be seen. The medulla oblongata was then drawn aside, and the roots of the nervus accessorius were carefully divided with scissors. No motions took place. The roots of the vagus were then cut, separately and singly from behind forwards; and at almost every section motion took place, either in the soft palate, or in the upper part of the pharynx, or in both at once. The experiment was repeated with every caution on five animals, and always gave the same results.

20. A calf's head was sawn longitudinally, and several roots, supposed to belong to the vagus being irritated, motions were produced in the pharynx and soft palate. Around every such root a silk thread was tied, and when the experiment was finished they were all traced to their origins. By far the greater part belonged to the vagus; one pair only came from the glosso-pharyngeus; but as experiment 17 shows that the latter nerve can excite motion in neither the upper part of the pharynx nor in the soft palate, it follows that the motions that were seen in this case must have depended on the vagus.

21. In another calf's head similarly prepared, the accessorius and glosso-pharyngeus were carefully removed as far as the foramen lacerum. The roots of the vagus were then galvanized, and motions took place in the superior and inferior constrictors of the pharynx, and in the crico-thyroideus, and in all the muscles of the soft palate. Subsequent dissection showed, that this experiment had been correctly performed.

22. Heads of calves, sheep, goats, cats, and dogs, were prepared directly after death, so as to expose the muscles of the larynx, pharynx, and soft palate. The roots of the vagus were then galvanized, and independent contractions ensued in the levator palati, azygos uvulæ, constrictor superior, c. inferior (but never in the c. medius), pharyngo-palatinus, and crico-thyroideus.

23. Different calves, sheep, goats, dogs, and cats were killed, by opening the carotids or abdominal aorta, and the descending branches of the vagus were exposed. Its roots were then irritated within the skull, and the most violent motions of the œsophagus instantly ensued, extending through its whole length, and exactly resembling those of voluntary muscles. The stomach never moved except at its cardiac portion by the communication of the movements of the œsophagus.

24. The larynx viewed from the spine during these experiments often presented manifest motion, but more rarely an alteration in the dimensions of the glottis, than a twitching of the prominent parts of the arytenoid cartilages. When the muscles were exposed the crico-arytenoideus posterius and lateralis were seen to move in the cat and calf, and the latter only in the dog; but the results of these experiments were often confused by the nerves being divided in the process of exposing the muscles; and it was probably on this account that the arytenoid muscles did not contract on irritating either the vagus or the accessorius.

25. After a cat had been killed by a stab in the heart, the vagi were divided below the superior laryngeal, and the glottis was exposed. Irritation of the superior laryngeal did not move the glottis, but on irritating the lower part of the nerve the glottis was widened, and sometimes the vocal ligaments were stretched. On irritating the superior laryngeal the exposed muscles that contracted were the constrictor faucium superior, the crico-thyroideus, and (in dogs and calves), the thyro-hyoideus. On irritating the lower part of the vagus, the muscles that contracted were the crico-arytenoideus posticus and lateralis.

26. In two young dogs the cerebrum and cerebellum were removed, the glottis being exposed it opened and shut with every effort of respiration. In one, the superior laryngeal nerves were divided, and the motions of the glottis were not in the least affected in consequence. In the other the vagi were divided, so as to destroy the influence of the recurrent, and the glottis remained permanently closed. The same result followed the division of the vagus within the skull in young dogs; but it is probable that this closure of the glottis is the consequence, not of any muscular contraction, but of the pressure of the atmosphere, which, when the muscles are paralyzed, forces the edges of the glottis together at every inspiration.

28. In different animals killed by bleeding, the chest and abdomen were opened, and the roots of the vagus galvanized, but no motion was seen in either stomach, intestines, heart, or trachea; or, at least, the increase of the peristaltic motions of the first was not more than would result from the contact of the air, or from the communication of the movements of the œsophagus.

29. No one of the muscles just mentioned as being influenced by the vagus could be made to contract through any other nerve. In experiments on nearly 100 animals, there were but two which seemed to indicate that the influence ascribed to the vagus belonged to other nerves; in one cat irritation of the accessorius excited motion of the soft palate, and in one calf that of the glosso-pharyngeus excited contractions of the crico-thyroideus, but it is quite possible that these anomalies were the results of error in the experiments.

VIII. *Nervus accessorius.* 30. When this nerve was galvanized within the skull in cats, dogs, goats, calves, and rabbits, violent motions ensued in the neck and shoulder, and when the skin was removed the muscles contracting were seen to be the whole of the sterno-cleido-mastoid and the trapezius; no motion was ever seen in the soft palate, (except in the one cat in No. 29), the pharynx or the larynx, but they were acted on as soon as the vagus was irritated.

31. The heart was exposed in a rabbit killed by hemorrhage; its contractions were but few and distant, and they did not in any degree correspond with the repeated irritation of the accessorius within the skull.

All these results occurred uniformly in a great number of experiments; the muscles of the larynx, pharynx, and soft palate, &c. were always moved when the vagus, but never when the accessorius was irritated; the excitement of the latter produced motions only in the trapezius and sterno-mastoid. Now since it is not at all probable, that in irritating the accessorius certain roots belonging to it were *always* missed, and that in irritating the vagus these same fibres were *always* included, there can, Volkmann thinks, be no doubt that the motor functions he has ascribed to the vagus do really belong to it, and not to the accessorius, and that the latter has no influence on the motions of the palate, fauces, and glottis.

IX. *Hypoglossal Nerve.* 34. This nerve always arises in several bundles of roots that pass through separate holes in the dura mater; and a ganglion being usually seated on one of them, has given rise to the general opinion that this is a nerve of mixed function. The ramus descendens has always been considered a branch of this nerve, but if the mode in which they are connected be examined, it will be found that all the fibres of the former do not pass centripetally into the trunk of the hypoglossal, but that some go on through it centrifugally, and therefore cannot be said to come from it. This is the case in man, calves, sheep, rabbits, and cats. In the horse, the author once saw that what is commonly called the descendens noni was really a ramus ascendens, receiving no fibres from the hypoglossal, but conveying all the fibres it contained to it. These fibres came from the two upper cervical nerves.—(See further the paper on Anastomoses of Nerves.)

35. In several fresh slain animals the hypoglossal was irritated at its root; motions ensued in the styloglossus, hyoglossus, genio-glossus, lingualis, and (in the calf), the hyo-epiglottis. In rabbits, irritation of either root excited contraction. Experiments were repeatedly made to see whether irritation of this nerve would produce motions of the omo-hyoideus, sterno-hyoideus, and sterno-thyroideus, but only the sterno-hyoideus could be made to move, and this in only two calves and one dog out of all the animals experimented on; facts which, with those mentioned in the preceding paragraph, are sufficient to prove that the hypoglossal gives but a few motor fibres to the descendens noni, and in general none but those for the thyro-hyoideus.

36. Each root of the hypoglossal was separately divided in a calf, and at every division a distinct motion of the tongue occurred. The little root with the ganglion however was overlooked; but in subsequent experiments, on galvinizing it, motion was repeatedly and regularly excited in a small space on the back of the tongue. The same experiment was twice repeated with every caution lest the galvanic action should be too powerful, but the result was in both the same.

X. *The cervical Nerves.* 37. These join the cerebral nerves in two different modes, their fibres pass into them either centrifugally or centripetally. The latter direction is generally disregarded, but repeated microscopic examinations have proved to the author, that branches of cervical nerves do thus pass to parts where they are least expected to be found. (This subject is treated of in a separate paper, on Anastomoses of Nerves.)

38. A sheep having been pithed between the occiput and atlas, the pharynx was exposed, and a galvanic current directed through the cervical part of the cord. Violent contractions ensued in the muscles of the neck, but none in the œsophagus, and this occurred repeatedly with very slight galvanic forces. There was indeed a slight undulatory motion of the œsophagus, such as the contact of the air might cause, but no general muscular action corresponding with the stimulus, or like that produced by irritating the vagus (23.). The same results followed irritation of the cervical nerves themselves where they are leaving the vertebral canal.

39. Irritation of the (motor) roots of the first cervical nerve excited motion in the sterno-hyoideus, sterno-thyroideus, and thyro-hyoideus (which was in one case moved by irritation of the hypoglossal also), but neither the sterno-mastoid nor the pharynx. Irritation of the second nerve excited several muscles in the neck, but none of those just mentioned, nor the œsophagus.

A number of similar experiments all tended to prove negatively that the movements of the pharynx and œsophagus are independent of the cervical nerves and governed by the vagi.

43. The muscles of the tongue were exposed in a fresh-slain sheep, and the descendens noni divided. At the instant of division the genio-hyoideus twitched, and this was repeated at every irritation. The central portion of the nerve was galvanized in several animals, and motions ensued in the hyo-glossus, genio-hyoideus, genio-glossus and lingualis, but it was difficult to say whether any of these, except the first, were moved primarily. Irritation of the root of the first cervical nerve regularly produced a motion of the tongue by which it was curved upwards, the lingualis being set in action.

45. As the general results of these experiments (which however are probably more valuable in their details,) the author gives the following conclusions:

A. All the nerves of the head, except the three of special sense, are motor (in a greater or less degree?).

B. Each muscle of the head (two of those of the eye excepted) receives its motor power from one nerve only, and therefore the voluntary and automatic motions of these muscles both depend on the same nerve.

C. This was found true of so many animals, that it is probably true of man also.

D. Some muscles of the tongue receive (as Valentin showed the iris does) branches from the cervical as well as from the cerebral nerves, a fact by which the defects of speech in disease of the cord are intelligible.

E. Motor nerves may have ganglia on their roots.

*Müller's Archiv. Heft* v. 1840.

---

*On Electrical Currents in the Nerves.* By Professor BISCHOFF of Heidelberg.

THIS paper contains a brief series of experiments performed by Professors Bischoff and Jolly, of which the results corroborate entirely the conclusions drawn by most other physiologists from the facts already known. They establish, first, that even the most delicate galvanometers can detect no current of electricity in the nerves; although they are such bad conductors of electricity, that its passage through them is not discernible even when its force is such as would act on very coarse galvanometers. These two facts together are conclusive against the existence of the supposed natural currents, since if they existed in such bad conductors, it is impossible but that the electrical tension would be sufficient to affect the galvanometer. But on the other hand, it is clearly proved that the nerves themselves are the most delicate of galvanometers, being so irritable to the electrical stimulus that muscular contractions are excited by a current too weak to be detected by the ordinary galvanometer-needle. Other experiments are alluded to which seem to prove that there is no free electricity in either the brain or spinal cord.

*Müller's Archiv. Heft* i. 1841, *p.* 20.

---

*On the Circulation in the Infusoria.* By Dr. ERDL of Munich.

THE author in a letter to Professor Müller says, "I have now very often seen and shown to my friends about here a kind of circulation in the infusoria, a phenomenon so remarkable that I cannot but wonder that it is not mentioned by any microscopic observer. I find it most distinctly in the Bursaria vernalis, whose abdomen, you know, appears to be quite full of green globules. Of these globules, those which lie near the periphery of the animal are incessantly moving (whether the animal itself be still or not) in an elliptic current upwards and downwards. In this current three or four globules always lie close by one another and move together with the stream. It has no relation whatever to the vivid ciliary motion that is constantly going on on the outer surface."

*Müller's Archiv. Heft* iii. 1841.

---

*On the Functions of the Anterior and Posterior Columns of the Spinal Cord.* By Dr. KUERSCHNER.

THE experiments here related, to establish that the anterior and posterior columns are respectively and exclusively motor and sensitive, are simple and founded on the well-known phenomena of reflex action.

The facts that individual muscles can be voluntarily moved, and that isolated sensitive impressions can be perceived, prove that the nervous principle is conducted along the spinal cord according to the same laws as it is along the nerves themselves. Reflex motions, therefore, cannot take place when the supposed sensitive columns are stimulated at the top of a divided cord, for the sensitive fibres cannot conduct centrifugally, nor can any motions take place in consequence of stimulating those columns unless they contain motor filaments. But in several experiments, chiefly performed on decapitated frogs, no motions whatever were excited by stimulating the upper part of the posterior columns, though by the slightest touch of the anterior severe convulsions were produced. The same result was obtained in whatever way the experiment was modified, so that there can be little doubt that there are no fibres in the posterior columns which are capable of conveying impressions (directly) to muscles.

The converse, namely, that there are no fibres in the anterior columns capable of conveying sensitive impressions, was proved in a similar manner. Thus, reflex motions occur only with the assistance of the centripetal nerves; and therefore when the posterior roots of the nerves of one of the legs of a frog are divided, irritation of the skin of that leg should cease to excite motion. And this in reality occurs; whenever the posterior columns, or roots of the nerves of a limb are destroyed, all reflex motion on irritating its skin is put an end to, although irritation of the anterior columns or roots still produces active muscular contractions.

*Muller's Archiv.* 1841. *Heft* i, p. 15.

